# Lifetime secondhand smoke exposure and childhood and adolescent asthma: findings from the PIAMA cohort

**DOI:** 10.1186/s12940-017-0223-7

**Published:** 2017-02-23

**Authors:** Edith B. Milanzi, Bert Brunekreef, Gerard H. Koppelman, Alet H. Wijga, Lenie van Rossem, Judith M. Vonk, Henriëtte A. Smit, Ulrike Gehring

**Affiliations:** 1 Institute for Risk Assessment Sciences (IRAS), Division of Environmental Epidemiology, Utrecht University, P.O. Box 80178, 3508 TD Utrecht, The Netherlands; 2 Julius Center for Health Sciences and Primary Care, University Medical Center Utrecht, Utrecht, The Netherlands; 3 Department of Pediatric Pulmonology and Pediatric Allergology, GRIAC Research Institute, University of Groningen, University Medical Center Groningen, Beatrix Children’s Hospital, Groningen, The Netherlands; 4 Groningen Research Institute for Asthma and COPD (GRIAC), University of Groningen, University Medical Center Groningen, Groningen, The Netherlands; 5 Centre for Prevention and Health Services Research, National Institute for Public Health and the Environment (RIVM), Bilthoven, The Netherlands; 6 Department of Epidemiology, University of Groningen, University Medical Center Groningen, Groningen, The Netherlands

**Keywords:** Secondhand smoke, Asthma, Longitudinal data, Environmental epidemiology

## Abstract

**Background:**

Secondhand smoke (SHS) exposure is a modifiable risk factor associated with childhood asthma. Associations with adolescent asthma and the relevance of the timing and patterns of exposure are unclear. Knowledge of critical windows of exposure is important for targeted interventions.

**Methods:**

We used data until age 17 from 1454 children of the Dutch population-based PIAMA birth cohort. Residential SHS exposure was assessed through parental questionnaires completed at ages 3 months, 1–8 (yearly), 11, 14, and 17 years. Lifetime exposure was determined as; a) time window-specific exposure (prenatal, infancy, preschool, primary school, and secondary school); b) lifetime cumulative exposure; c) longitudinal exposure patterns using latent class growth modeling (LCGM). Generalized estimation equations and logistic regression were used to analyze associations between exposure and asthma at ages 4 to 17 years, adjusting for potential confounders.

**Results:**

With all three methods, we consistently found no association between SHS exposure and asthma at ages 4 to 17 years e.g. adjusted overall odds ratio (95% confidence interval) 0.67 (0.41–1.12), 1.00 (0.66–1.51) and 0.67 (0.41–1.11) for prenatal maternal active smoking, infancy, and preschool school time window exposures, respectively.

**Conclusion:**

We assessed lifetime SHS exposure using different methods. Different timing and patterns of SHS exposure were not associated with an increased risk of asthma in childhood and adolescence in our study. More longitudinal studies could investigate effects of lifetime SHS exposure on asthma in adolescence and later life.

**Electronic supplementary material:**

The online version of this article (doi:10.1186/s12940-017-0223-7) contains supplementary material, which is available to authorized users.

## Background

Exposure to secondhand smoke (SHS) along with exposure to other environmental, lifestyle and genetic factors has been found to be associated with asthma [1–3]. Since asthma is one of the most common chronic respiratory diseases and most asthma begins in childhood and adolescence, exposure to SHS during these periods is of particular interest [4].

Prenatal and early life postnatal exposure to SHS has been found to be associated with an increased risk of asthma during the first 10 years of life [2, 5–8]. Studies of the association of asthma with SHS exposure later in life and studies of the association between lifetime SHS exposure and adolescent asthma are scarce. Consequently, the relevance of SHS exposure later in life and for adolescent asthma is largely unknown. To date, four prospective studies [6, 9–11] have assessed the association of pre and postnatal SHS exposure on asthma in adolescence but results are inconsistent. Only one study out of these considers SHS exposure throughout childhood.

Longitudinal cohort studies seldom pay attention to patterns of exposure over time. Exposure to SHS during different time windows may also differentially affect the presence of asthma, therefore knowledge of critical time windows of exposure is important in implementing targeted interventions. Therefore, in this study, we aim to use three methods to determine the role of timing of SHS exposure and investigate if there is a critical time window of SHS exposure that contributes to asthma up to adolescence, and to examine cumulative exposure and detailed longitudinal patterns of exposure from repeated measures and investigate their associations with asthma prevalence at age 17.

## Methods

### Study population and design

We obtained data from the Dutch population-based PIAMA (Prevention and Incidence of Asthma and Mite Allergy) birth cohort that started with 3963 children born in 1996/1997 [12]. Questionnaires were completed by parents during pregnancy, 3 months after birth, then yearly from age 1 to 8; and at ages 11, 14 and 17 by both, parents and children. Questionnaires comprised questions on SHS exposure and asthma symptoms, diagnoses and medication as well as socio-demographic characteristics, parental atopy, and other environmental exposures and lifestyle characteristics.

Our primary study population consists of all adolescent children with complete data on both asthma at age 17 and SHS exposure from pregnancy until age 17 (*N* = 1454, Additional file 1: Figure S1).

### Ethics statement

Ethical approval was obtained from authorized institutional review boards. Children’s parents or legal guardians and children themselves provided written informed consent.

### SHS exposure assessment

We assessed SHS exposure through the repeated parental questionnaires from pregnancy till age 17. Exposure at age 17 was assessed using the adolescent questionnaires if the participant had moved out of his/her parents’ home (*N* = 50). Postnatal residential SHS exposure was assessed by reports of anybody smoking inside the home (yes; yes, but less than once a week; never). In addition, the number of cigarettes smoked per day for those who answered ‘yes’ was obtained, and at ages 1–4, information on smoke exposure outside child’s home if children regularly spent at least half a day outside their home.

Lifetime SHS exposure was determined using three methods: First, we distinguished five *time windows.* To optimize the implementation of potential preventive measures during a specific time window we chose time windows for ages that match appropriate settings for prevention in the Netherlands: *prenatal* period (pregnancy), *infancy* time window (3 months after birth) corresponding to prevention in well-baby clinics, *preschool* time window (1–4 years) for prevention through infant health care, and *primary school* (5–11 years) and *secondary school* (>12 years) time windows for prevention in primary and secondary school, respectively. Time window-specific exposures were assessed as detailed in Additional file 1: Figure S2. In brief, for the prenatal period, four categories were defined: maternal active smoking, maternal sometimes passive smoking (>4 h per day), maternal rare passive smoking (1–4 h per day), and never (no active or passive smoking). Exposure during infancy was defined by the 3-month questionnaire using the original categories. Exposure during preschool, primary school and secondary school time windows was determined through categories created from two to five questionnaires: if the response to the question on anyone smoking in the house was ‘yes’ for the whole time window, children were classified as ‘always exposed’; if the response was ‘yes’ at least once during the time window, children were classified as ‘sometimes exposed’; if the response was ‘yes, but less than once a week’ at least once and ‘never’ otherwise, children were classified as ‘rarely exposed’; if the response was ‘never’ during whole time window, children were classified as ‘never exposed’.

Second, *lifetime cumulative SHS exposure* was defined based on the well documented dose –response relationship of SHS and asthma [13] by assigning points to questionnaire responses on SHS exposure (prenatal exposure: maternal active smoking = 2 points, any maternal passive smoking = 1 point; neither active nor passive smoking = 0 points; postnatal exposure for each questionnaire: yes = 2 points; yes, but less than once a week = 1 point; never = 0 points) and summing score points for all questionnaires from pregnancy to age 17. The score ranged from 0 (no SHS exposure) to 26 points (highest exposure at all 13 follow-ups). Children with SHS exposure were divided into three categories of equal size defined as passive low (scores 1–3), medium (scores 4–12) and high (scores 13–26).

Third, in order to combine exposure during different time windows and account for cumulative exposure, we used a data-driven approach known as latent class growth modeling [14] (LCGM, TRAJ procedure in SAS 9.4, Cary, USA) to define lifetime *longitudinal patterns of exposure* from pregnancy to age 17. These patterns reflect the different underlying subpopulations existent within the whole population based on the probability of being exposed to SHS over time. The procedure allocates individuals based on the probability of belonging to a particular pattern taking into account the status of exposure at each time point and translates it into a cumulative pattern over time. The higher the probability, the higher the likelihood of being allocated to that particular pattern. SHS exposure was dichotomized at each time point for this procedure (any exposure, yes/no). To determine the number and shape of patterns of SHS exposure in the population, we first assumed that there is one homogeneous non-changing pattern of SHS exposure from pregnancy till age 17, by specifying the intercept only. We then further investigated if there was more than one pattern and different pattern shapes by including more groups and higher order polynomials. We repeated this procedure for models assuming up to five groups of patterns for polynomials up to the order of three (models with six or more patterns did not converge). All models were compared and the best model was defined as one with smallest BIC (Bayesian Information Criterion).

### Asthma definition

Asthma at ages 4 to 17 years was defined by the presence of two out of the following three criteria based on parental questionnaires: wheezing in the past 12 months, doctor diagnosed asthma ever, and prescription of asthma medication in the past 12 months according to the MeDALL protocol [15]. We also defined asthma phenotypes based on age of onset and persistence as follows: “early transient” defined as any asthma according to the definition described above between 4 and 6 years but not later, “persistent” defined as asthma between 4 and 6 and between 14 and 17 years; “intermediate onset” defined as first asthma between 7 and 11 years, “adolescent onset” defined as first asthma between 14 and 17 years; and never defined as no report of asthma at ages 4–17 years.

### Statistical analyses

We used Generalized Estimating Equations (GEE) to analyze associations of asthma at ages 4 to 17 years with SHS exposure during the prenatal, infancy and preschool time windows; multiple logistic regression models to analyze associations of asthma at age 17 with SHS exposure during the primary and secondary school time windows, as well as cumulative scores and the longitudinal patterns of exposure; and polytomous logistic regression to analyze associations of asthma phenotypes with exposure during the prenatal, infancy and preschool time windows. Age-specific estimates of associations with exposure during prenatal, infancy and preschool time windows were obtained from GEE models with exposure-age interaction terms. In analyses of exposure patterns, the pattern variables were included as exposure variables in the model. Observations were weighted according to posterior probabilities of belonging to a particular pattern of exposure to account for uncertainty in allocation of individuals to patterns.

Adjusted and unadjusted analyses were performed adjusting for the following potential confounders identified from literature and prior knowledge: parental education (defined as maximum of either mother’s or father’s education; low, medium, high), gender, parental atopy, breastfeeding (>12 weeks: yes/no), having older siblings (yes/no), maternal age at birth (continuous), active smoking (smoking at least once a week at 14/17 years), resident region at birth (north, middle, west). Time-varying confounders such as presence of pets (yes/no), gas cooking (yes/no), presence of dampness and moulds (yes/no), and overweight (yes/no/unknown based on BMI using the International Obesity Task Force gender-specific cut-off points [16]) were selected from the earliest available questionnaire. Exposure to ambient air pollution was estimated by land-use regression modeling [17] and defined as annual average nitrogen dioxide (NO_2_) concentration at the home address at birth.

We performed a number of sensitivity analyses. In order to investigate the possible effect of selecting children with complete SHS data, we repeated analyses with extended populations; i.e. children with complete exposure information for a specific time window instead of from birth till age 17 for time window-specific exposures and individuals with asthma data, but incomplete SHS exposure data (*N* = 1871) for the longitudinal patterns. We repeated adjusted analyses with time-varying confounders defined based on the latest available questionnaire (14/17 years). We also excluded active smokers from all analyses and additionally adjusted for low birth weight, which could be on the causal pathway between asthma and SHS exposure. Modifications of the association between SHS and asthma by parental atopy, presence of pets, gender and parental education have been suggested [18–23] and were explored in stratified analyses.

All analyses were performed with SAS version 9.4. Statistical significance was defined by a two-sided alpha of 0.05.

## Results

Study population characteristics are presented in Table 1. Half of the children were boys, 60% had highly educated parents, and 52% had atopic parents. Parents who reported any SHS exposure from pregnancy till age 17 (*N* = 823) were less often atopic and less often highly educated than parents who did not report any exposure (Additional file 1: Table S1). Asthma prevalence ranged from 5% at age 17 to 8% at age 4 (Additional file 1: Figure S3). Prevalences were 7, 3, 4 and 1% for early onset, intermediate, persistent, adolescent onset asthma. Baseline characteristics were similar for the study population and the excluded population except for higher prevalence of high parental education and breastfeeding and a lower prevalence of pet ownership in the study population (Additional file 1: Table S2).

**Table 1:** Characteristics of study population (*N* = 1454)

Characteristics	n/N	(%)
Parental atopy (Yes)	749/1454	51.5
Gender (Boys)	721/1454	49.6
Presence of pets at 3 months (Yes)	674/1454	46.3
Presence of moulds at 1 year (Yes)	386/1454	26.5
Breastfeeding >12 weeks (Yes)	772/1449	53.3
Overweight at 3 years		
Yes	95/1454	6.5
No	1122/1454	77.1
Unknown	237/1454	16.3
Gas cooking (Yes)	1204/1448	83.1
Older siblings (Yes)	727/1454	50.0
Parental education		
Low	125/1454	8.6
Intermediate	464/1454	31.9
High	864/1454	59.5
Region		
North	454/1454	31.1
Middle	633/1454	43.6
Western	367/1454	25.2
Ethnicity (Dutch)	1322/1435	92.1
Active smokers (14/17 years)	193/1454	13.2
	N (Mean (Range))	
Maternal age at birth (years)	1438 (31.1 (18–42))	
Outdoor NO_2_ at home address at birth (μg/m^3^)	1448 (22.8 (9.2–59.6))	

**Fig. 1:**
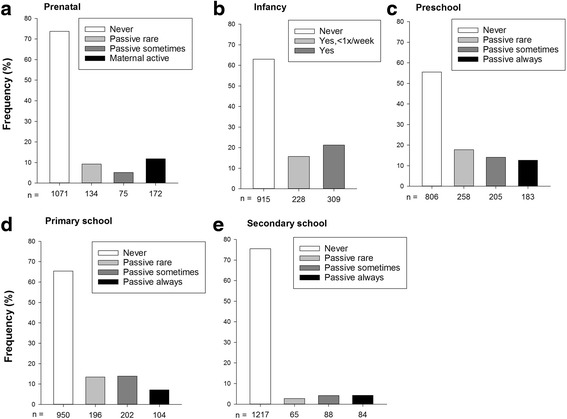
Frequency distribution of time-window-specific SHS exposure

A decreasing trend in ETS exposure was noted. Of the 1454 children, 11% were exposed prenatally through maternal active smoking. Seven and 4% of the children were always passively exposed during primary school and secondary school time windows, respectively (Fig. 1). In the secondary school time window, 13% of the children had taken up smoking themselves. The number of cigarettes smoked per household was small (median number ranged from 5 to 10 cigarettes per day from pregnancy till age 17) and SHS exposure outside the child’s home was infrequent (6–7% of the participants were sometimes exposed and 2% always exposed outside the home at ages 1 to 4 years) and was therefore not considered in the analysis.

**Fig. 2:**
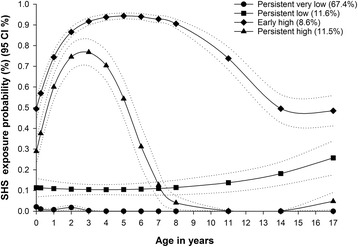
Longitudinal patterns for SHS exposure from birth until age 17

A total of 631 children had a sum score of 0 points, 24 children were assigned the maximum number of 26 points. Four distinct longitudinal patterns of SHS exposure patterns from pregnancy to age 17 were identified: “persistent very low” (67.4%) representing individuals with very low probability of exposure throughout the follow-up; “persistent low” (11.6%) representing children with a low probability of exposure throughout the follow-up; “early high” (8.6%) representing children with a high probability of exposure from birth until around age 8; and “persistent high” (11.5%) representing children with a high probability of exposure during almost the entire follow-up (Fig. 2). The correlation between the different exposure variables was low to moderate ranging from 0.29 for the correlation between the secondary school time window exposure and cumulative scores to 0.73 for the correlation between the preschool time window and the cumulative scores.

**Fig. 3:**
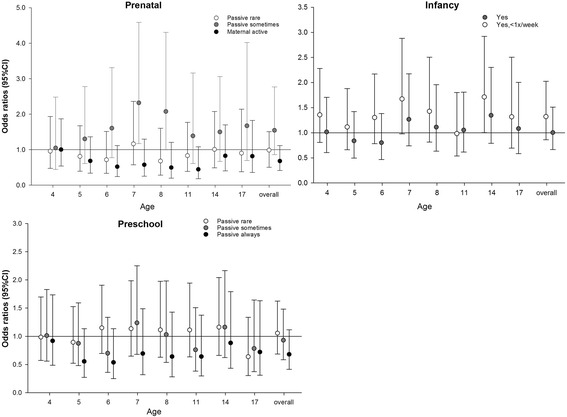
Adjusted overall and age-specific association of SHS exposure with asthma at ages 4 to 17 for prenatal, infant and preschool time-window-specific exposures. Adjusted for gas cooking at 3 months, overweight at 3 years, presence of pets at 3 months, presence of molds at 1 year, outdoor NO_2_ exposure at home address at birth, gender, active smoking, breastfeeding, older siblings at birth, parental atopy, parental education, region and maternal age

**Fig. 4:**
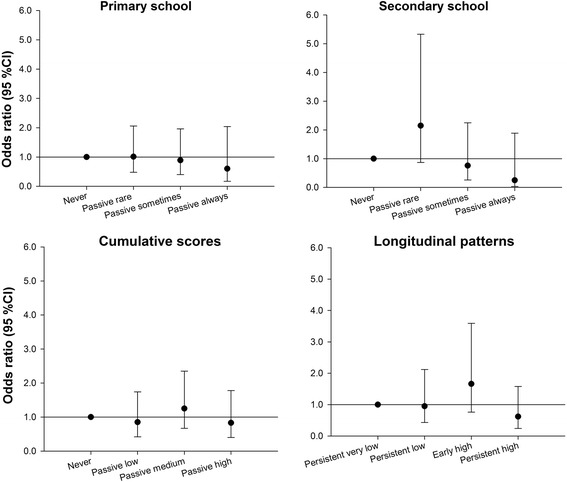
Adjusted association of SHS exposure with asthma at age 17 for primary and secondary school time windows and lifetime exposures. Adjusted for gas cooking at 3 months, overweight at 3 years, presence of pets at 3 months, presence of molds at 1 year, outdoor NO_2_ exposure at home address at birth, gender, active smoking, breastfeeding, older siblings at birth, parental atopy, parental education, region and maternal age

Adjusted associations between the different methods of SHS exposure assessment and asthma are presented in Figs. 3 and 4. Unadjusted odds ratios (Additional file 1: Table S3) and adjusted odds ratios for the overall associations were generally similar. There were no statistically significant associations between asthma at ages 4 to 17 years and early life time windows exposure except for an increased risk of asthma at ages 7 and 8 years in children of mothers who were sometimes exposed to maternal passive smoking during pregnancy. Similarly, we did not see associations at age 17 for the primary and secondary school exposures, cumulative exposures, and longitudinal patterns of exposure. In the same line, we did not observe any significant associations between asthma phenotypes and SHS exposure (Additional file 1: Table S4).

In sensitivity analyses, extending populations did not change results for the time windows as well as the associations between asthma and patterns (Additional file 1: Figures S4–S6). Similarly, defining time-varying confounders based on data from the most recent questionnaire or additional adjustment for low birth weight did not influence results (Additional file 1: Figures S7 and S8). We also did not see any changes from the main analysis when we excluded active smokers (Additional file 1: Figure S9).

Stratified analyses by parental atopy, presence of pets, gender, and parental education did not provide any evidence for a modification of the association between SHS and asthma by these factors. Additional file 1: Figures S10–S13 show results of these stratified analyses for the preschool time window, results for the prenatal and infancy windows were similar.

## Discussion

We characterized lifetime SHS exposure using three different methods: by time windows, by cumulative scores, and by longitudinal patterns. With none of the methods, we observed an association with asthma in childhood and adolescence in this cohort of children followed since pregnancy.

Many studies have focused on exposure during pregnancy as well as infancy [2, 6, 11] and attempts have been made to combine time point specific exposures to characterize cumulative SHS exposure in longitudinal studies [24]. To our knowledge, there is limited literature on characterizing lifetime SHS exposure using data from every follow-up in longitudinal studies with longer follow-up periods. In this study, we used three different methods using data from all 13 follow-ups. Each of these methods covers a different aspect of lifetime exposure: Time-window-specific analyses were used to determine the role of the timing of exposure; cumulative scores were used to quantify cumulative SHS exposure throughout the follow-up, and longitudinal patterns described lifetime SHS exposure patterns including changes in exposure over time. Four patterns were deduced in our population: persistent very low, persistent low, early high and persistent high exposure.

Few other studies have assessed associations between prenatal and postnatal SHS exposure with asthma during adolescence [6, 10, 11]. A longitudinal study from Australia [6] found a positive association between maternal active smoking during pregnancy and asthma in 14 year-olds [OR (95% CI) 1.84 (1.16–2.92)]. The MUSP study, another Australian cohort [10] observed positive associations of prenatal and postnatal (at 6 months) maternal heavy smoking (≥20 cigarettes/day) and asthma at age 14 in girls [OR (95% CI) 1.98 (1.25–3.33)], but not in boys. No associations were found with lower numbers of cigarettes smoked in that study. Stratified analysis by gender in our study did not reveal any differences in the association between SHS exposure and asthma between boys and girls. Thacher et al. [11] explored associations of prenatal (maternal active) smoking and postnatal parental smoking with asthma in the first 16 years of life in the Swedish BAMSE cohort. Associations of prevalent and incident asthma until age 16 with SHS exposure in pregnancy, infancy (2 months after birth) were investigated adjusting for parental smoking throughout childhood. Asthma until age 4, but not at later ages, was associated with maternal smoking during pregnancy. We did not observe such an association between early SHS exposure and asthma at age 4 in the age specific analyses, nor in the analyses with asthma phenotypes in our study. The lack of association between parental smoking throughout childhood and asthma at age 16 in the Swedish cohort, however, is consistent with our findings. The generally low numbers of cigarettes smoked in the present study as compared to the Australian studies may explain the difference between the present study and the two Australian studies. The two mentioned Australian studies defined maternal smoking as smoking at any stage of pregnancy, which is comparable to our definition of smoking in at least the first 4 weeks of pregnancy and therefore, differences in exposure definitions likely do not explain the lack of association in our study. In contrast to our study, which assessed lifelong SHS exposure from birth until age 17, most of these prospective studies focused on exposure at one or few specific ages. Cross-sectional studies investigating the relationship between SHS exposure and adolescent asthma have also been conducted. Findings of these studies are as conflicting. A cross-sectional study from Hong Kong observed associations between current SHS exposure and asthma symptoms in 15 year-old adolescents [OR (95% CI) 1.45 (1.17–1.81)] [25]. In contrast, two other cross-sectional studies [26, 27] did not find significant associations between current SHS exposure and current asthma in children aged 11–15 years. However, cross-sectional studies have major limitations; recall bias may occur when assessing past exposures and a temporal relationship with asthma cannot be established. Apart from the Swedish study, none of these studies investigated lifetime SHS exposure and its relationship with asthma in adolescence. Our results are therefore not directly comparable to the results from the above mentioned studies.

We consider the use of different methods to define lifetime SHS exposure that cover different aspects of exposure i.e. lifetime exposure or the importance of the timing of exposure as the major strength of our study. Longitudinal trajectories are also useful descriptive tools in characterizing the population and identifying detailed patterns. SHS exposure data has not been extensively studied in this way to deduce distinct patterns. Other researchers can use this method with other exposures to detect patterns and explore their relationship with an outcome of interest. The prospective nature of the study also allowed small liability of recall bias from parental reports of SHS exposure.

Our findings should be interpreted considering the following limitations: We acknowledge that the cumulative scores were assigned arbitrarily, therefore, we cannot rule out that the use of the scores may have resulted in exposure misclassification. However, the choice of the method was based on the well documented dose –response relationship between SHS exposure and health outcomes including asthma and scores were categorized using tertiles as cut-offs. Therefore we believe that bias is no major concern. The number of cigarettes smoked was small in our population and decreased over time. As such we cannot rule out adverse effects of heavy smoking on asthma up to adolescence. The emphasis by health care providers on the health risks associated with SHS exposure may explain the observed decrease in SHS exposure prevalence over time however this is not a unique phenomenon to our study as prevalence of smoking has generally decreased in the Netherlands. In addition, in our study population, there were more atopic parents in the non-exposed group than in the exposed group. The reason for this may be that atopic parents may already smoke less than non-atopic parents in pregnancy and that children of atopic parents have a genetically increased risk of asthma and that they may be less likely to be exposed as parents of asthmatic children have been found to be more inclined to smoke less because of their child’s asthma [28]. We, therefore, investigated the potential modification of the SHS effect by parental atopy, but did not find any differences in the association between children born to atopic and non-atopic parents. Absence of exposure due to parental atopy is, therefore, unlikely the explanation of the lack of association between SHS exposure and asthma in our study. Another potential explanation for the lack of association could be the use of parental self-reported data, which could lead to differential exposure misclassification as parents of asthmatic children may tend to underreport their household smoking because of knowledge on the harmful consequences of SHS exposure. A validation study comparing SHS exposure self-reports with measured air nicotine levels in a subset of the PIAMA population, however, suggests self-reported information about SHS exposure generally provides valid estimates of residential exposure [29]. Therefore, the reasons for the lack of association between lifetime SHS exposure and asthma in our study remain unclear. However, more longitudinal studies could investigate effects of lifetime secondhand smoke exposure on asthma in later life.

## Conclusion

We investigated associations of the timing of secondhand smoke exposure as well as secondhand smoke exposure patterns from birth till age 17 with asthma till age 17. Asthma was not associated with any of the exposure metrics in this study.

## Additional file

Additional file 1: Type of data: Figures and tables. (PDF 1647 kb)

## Abbreviations

SHS: Secondhand smoke
PIAMA: Prevention and Incidence of Asthma and Mite Allergy
LCGM: Latent Class Growth Modelling
OR: Odds ratio
CI: Confidence interval
