# Supplementary material for: Lifetime secondhand smoke exposure and childhood and adolescent asthma: findings from the PIAMA cohort
Source: Environ Health. 2017 Feb 23;16:14. doi: 10.1186/s12940-017-0223-7 (PMC5324208; doi:10.1186/s12940-017-0223-7)
Supplement: Additional file 1: — Type of data: Figures and tables. (PDF 1647 kb) [file 12940_2017_223_MOESM1_ESM.pdf]

Lifetime secondhand tobacco smoke exposure and childhood and adolescent asthma: Findings from the PIAMA cohort

Edith B Milanzi, Bert Brunekreef, Gerard H Koppelman, Alet H. Wijga, Lenie van Rossem, Judith M Vonk, Henriëtte A Smit, Ulrike Gehring

Online supplement

Table S1. Baseline characteristics comparisons: exposed vs non-exposed participants **(N=1,454)** *

**Characteristic**	**Exposed (N=823)**		**Non-Exposed (N=631)**		**P-value**
	**N**	**%**	**N**	**%**	
Parental atopy (Yes)	383/823	46.5	366/631	58.0	<0.000
Gender (Boys)	407/823	49.4	314/631	49.7	0.907
Presence of pets at 3 months (Yes)	439/823	53.4	235/631	37.2	<0.000
Presence of moulds at 1 year(Yes)	217/823	26.3	169/631	26.7	0.858
Breastfeeding >12 weeks (Yes)	385/819	47.0	387/631	61.4	<0.000
Overweight at 3 years					
Yes	56/823	6.8	39/582	6.1	0.239
No	622/823	75.5	500/582	79.2	
Unknown	145/823	17.6	92/582	14.5	
Gas cooking at 3 months (Yes)	670/821	81.6	534/627	85.1	0.073
Older siblings (Yes)	395/823	48.0	332/631	52.6	0.080
Education	823		630		<0.000
Low	99	12.1	26	4.1	
Intermediate	296	35.9	168	26.6	
High	428	52.0	436	69.2	
Region	823		631		0.08
North	256	31.1	198	31.3	
Middle	342	41.5	291	46.1	
Western	225	27.3	142	22.5	
Ethnicity (Dutch)	745/814	91.5	577/621	92.9	0.332
Active smokers (14/17 years)	144/823	17.5	49/631	7.7	<0.000
	**N**	**Mean (Range)**	**N**	**Mean (Range)**	
Maternal age (years)	812	31.0 (18-42)	626	31.2 (21-42)	0.320
NO_2 at home address at birth (µg/m^3)	819	22.8 (9.2-59.6)	629	22.7 (9.5-45.1)	0.604

* Groups were compared using Chi-square tests for categorical variables and t-tests for continuous variables

Exposed= any positive response of SHS any exposure during any follow up, Non-exposed= no positive response of exposure on all follow ups.

Table S2. Baseline characteristics comparisons: Baseline population compared with the study population.*

	**Baseline population (N=3,963)**		**Study population (N=1,454)**		
**Characteristic**	**N**	**%**	**N**	**%**	**P-value**
Parental atopy (Yes)	2038/3963	51.4	749/1454	51.5	0.954
Gender (Boys)	2054/3963	51.8	721/1454	49.5	0.143
Presence of pets at 3 months (Yes)	2024/3941	51.3	674/1454	46.3	0.001
Presence of moulds at 1 years (Yes)	1047/3963	26.4	386/1454	26.5	0.924
Breastfeeding >12 weeks (Yes)	1703/3963	43.7	772/1449	53.2	<0.000
Overweight at 3 years					<0.000
Yes	206/3963	5.1	95/1454	6.5	
No	2765/3963	69.8	1122/1454	77.1	
Unknown	992/3963	25.1	237/1454	16.3	
Gas cooking (Yes)	3247/3963	82.7	1204/1448	83.1	0.742
Older siblings (Yes)	1986/3963	50.1	727/1454	50.0	0.908
Education					<0.000
Low	484/3812	12.7	125/1453	8.6	
Intermediate	1420/3812	37.2	464/1453	31.9	
High	1908/3812	50.1	864/1453	59.5	
Region					0.015
North	1231/3963	31.1	454/1454	31.2	
Middle	1586/3963	40.0	633/1454	43.5	
Western	1146/3963	28.9	367/1454	25.2	
Ethnicity (Dutch)	3327/3684	90.3	1322/1435	92.1	0.043
Active smokers(14/17 years)	216/1639	13.1	193/1454	13.2	<0.000
	**N, Mean (Range)**		**N, Mean (Range)**		
Maternal age at birth (years)	3871, 30.3 (17-42)		1438, 31.1 (18-42)		0.03
NO_2 at home address at birth (µg/m^3)	3937, 23.2 (8.7-59.6)		1448, 22.8 (9.2-59.6)		

* Groups were compared using Chi-square tests for categorical variables and t-tests for continuous variables

Table S3. Unadjusted (N=1,454) odds ratios (OR) with 95% confidence intervals (CI) for the association between tobacco smoke exposure and asthma until age 17*

	**Unadjusted OR (95% CI)**
**Prenatal**	
Never	Ref
Passive rare	0.97 (0.63-1.47)
Passive sometimes	0.91 (0.58-1.42)
Maternal active	0.73 (0.45-1.17)
**Infancy**	
Never	Ref
Yes, <1x/week	1.26 (0.81-1.94)
Yes	1.07 (0.73-1.56)
**Preschool**	
Never	Ref
Passive rare	0.88 (0.51-1.52)
Passive sometimes	1.55 (0.83-2.85)
Passive always	0.70 (0.43-1.15)
**Primary school**	
Never	Ref
Passive rare	0.94 (0.47-1.90)
Passive sometimes	0.72 (0.34-1.55)
Passive always	0.52 (0.16-1.70)
**Secondary school**	
Never	Ref
Passive rare	2.12 (0.88-5.13)
Passive sometimes	0.99 (0.35-2.80)
Passive always	0.22 (0.03-1.64)
**Cumulative scores**	
Never	Ref
Passive low	0.78 (0.40-1.57)
Passive medium	1.17 (0.65-2.12)
Passive high	0.71 (0.34-1.47)
**Longitudinal patterns**	
Persistent very low	Ref
Persistent low	0.74(0.28-1.96)
Early high	1.63 (0.77-3.42)
Persistent high	0.60 (0.25-1.44)

* Odds ratio estimates reported for prenatal, infancy and preschool times windows are longitudinal overall point estimates. Primary school, secondary school , cumulative scores and longitudinal patterns estimates are for asthma at age 17.

Table S4. Adjusted (N=1,454) odds ratios (OR) with 95% confidence intervals (CI) for the association between SHS exposure and asthma phenotypes$^{\gamma}$

	OR (95% CI)	OR (95% CI)	OR (95% CI)
**Prenatal***	**Passive smoking**	**Maternal active**	
Persistent	0.95 (0.45 - 1.99)	0.40 (0.13 - 1.15)	
Adolescent onset	0.63 (0.18 - 2.25)	1.01 (0.33 - 3.13)	
Intermediate	1.39 (0.61 - 3.15)	0.83 (0.28 - 2.44)	
Early transient	1.28 (0.72 - 2.25)	1.75 (0.99 - 3.08)	
**Infancy**	**Yes, <1x/week**	**Yes**	
Persistent	1.70 (0.88 - 3.27)	1.22 (0.64 - 2.32)	
Adolescent onset	0.61 (0.17 - 2.12)	0.98 (0.38 - 2.47)	
Intermediate	1.35 (0.61 - 2.98)	1.39 (0.64 - 3.03)	
Early transient	0.87 (0.48 - 1.58)	0.98 (0.59 -1.64)	
**Preschool**	**Passive rare**	**Passive sometimes**	**Passive always**
Persistent	1.02 (0.52 – 1.99)	0.81 (0.36 – 1.82)	0.60 (0.24 – 1.51)
Adolescent onset	0.36 (0.08 -1.61)	1.20 (0.42 -3.42)	1.27(0.43 - 3.72)
Intermediate	1.93 (0.93 -4.00)	1.29 (0.50 – 3.30)	2.18 (0.87 – 5.46)
Early transient	0.65 (0.34 -1.22)	1.29 (0.74 – 2.24)	1.16 (0.63 – 2.13)

γ : Odds ratios are interpreted in reference to the 'Never' exposed group and to 'Never' asthma phenotype.

*: Passive smoking categories combined in the prenatal time window due to low frequency cells. Adjusted for gas cooking at 3 months, overweight at 3 years, presence of pets at 3 months , presence of molds at 1 year , outdoor NO_2 exposure at home address at birth, gender, breastfeeding, active smoking, older siblings at birth, parental education, region and maternal age.

Figure S1. Breakdown of available SHS exposure information until age 17 for children with data on asthma until age 17

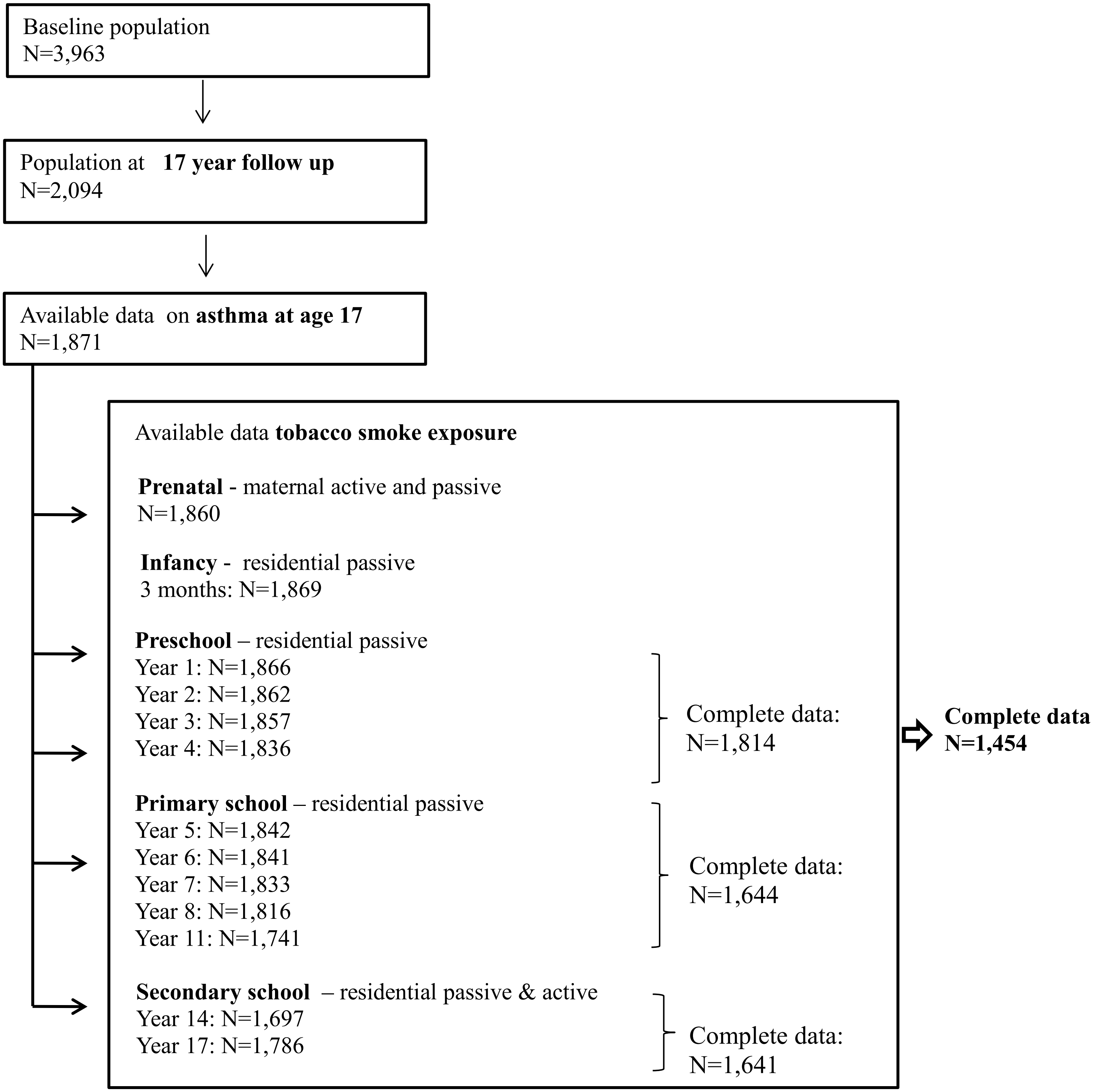

Figure S2. Flowcharts describing the definition of SHS exposure categories for the different time windows

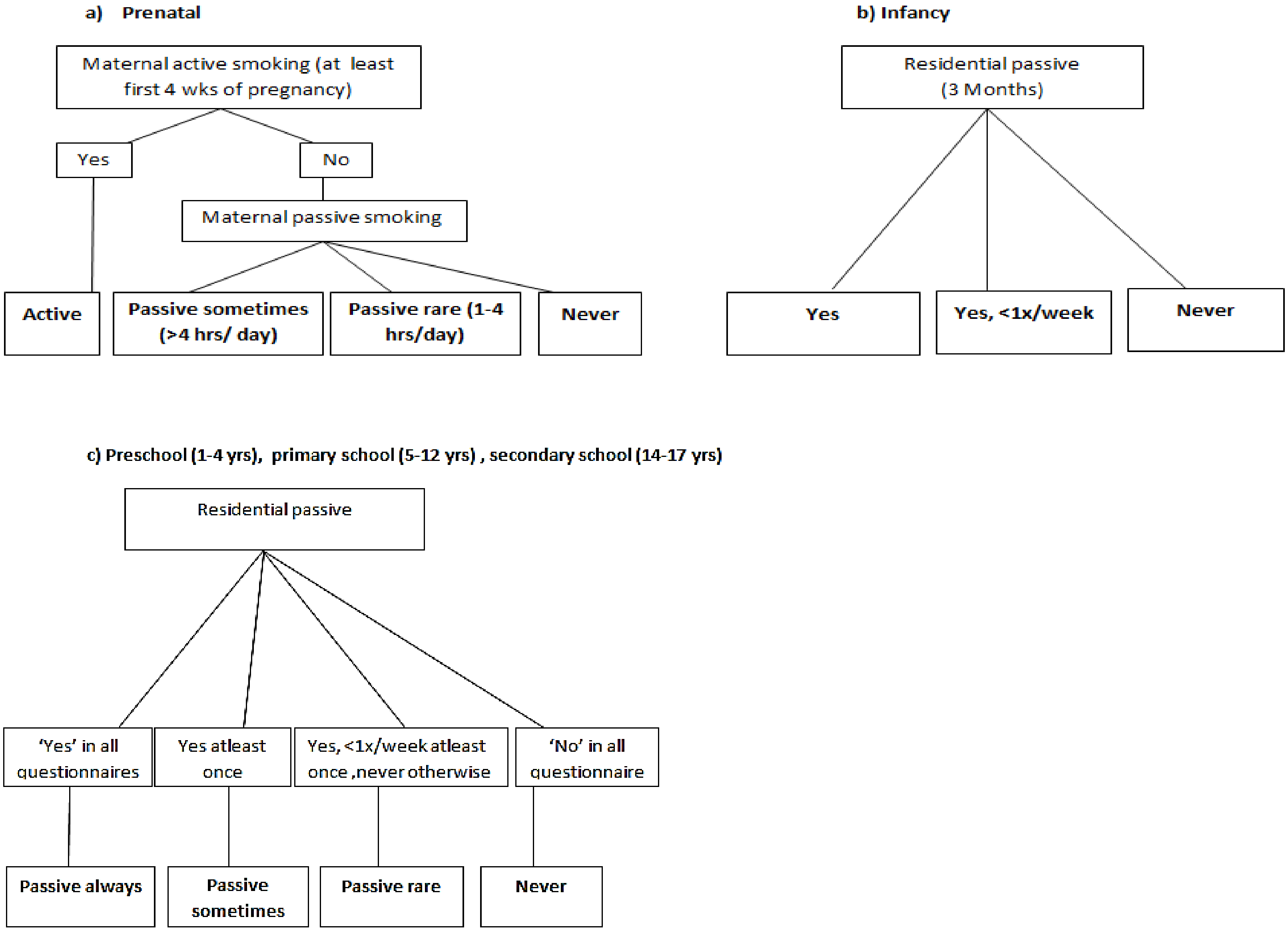

Figure S3 : Prevalence of asthma from age 4 to age 17.

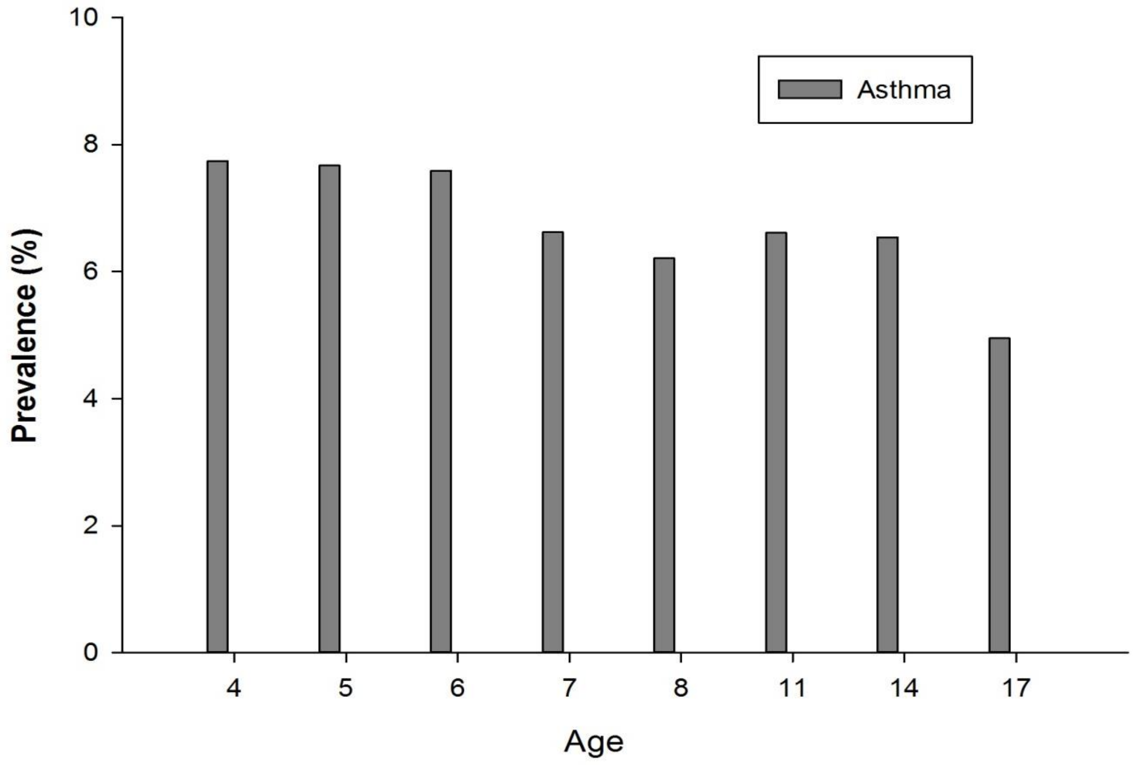

Figure S4. Adjusted association of SHS exposure with asthma until age 17 for extended populations per time window [¥]

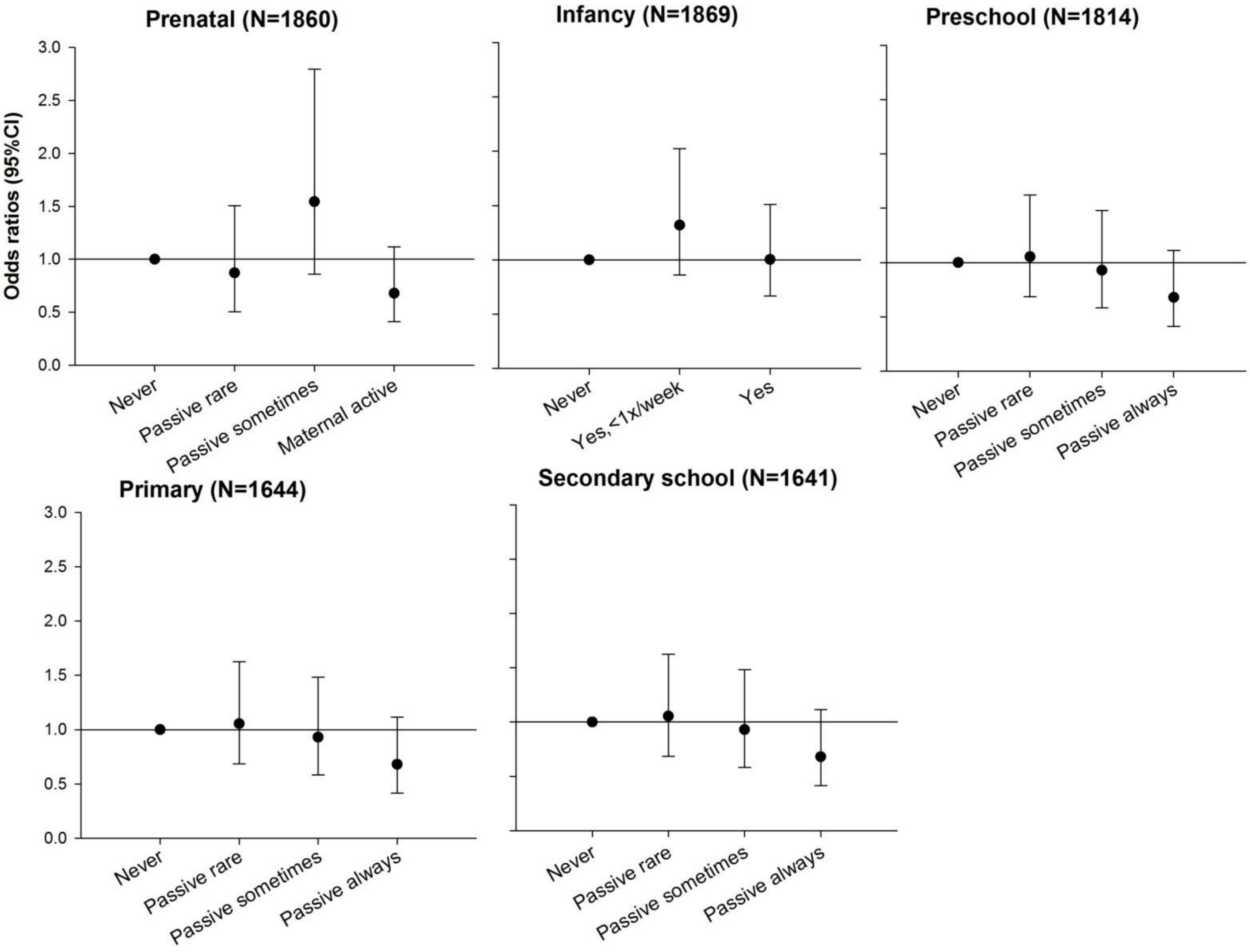

¥ Associations are overall associations with asthma at ages 4 to 17 years from GEEs for prenatal, infancy and preschool exposure, associations with asthma at age 17 from cross-sectional analyses for primary and secondary school windows. Adjusted for gas cooking at 3 months, overweight at 3 years, presence of pets at 3 months, presence of molds at 1 year, outdoor N02 exposure at home address at birth, gender, active smoking, breastfeeding, older siblings at birth, parental atopy, parental education, region and maternal age.

Figure S5. Longitudinal patterns for extended population of children with asthma data but incomplete SHS exposure data. (N=1871)

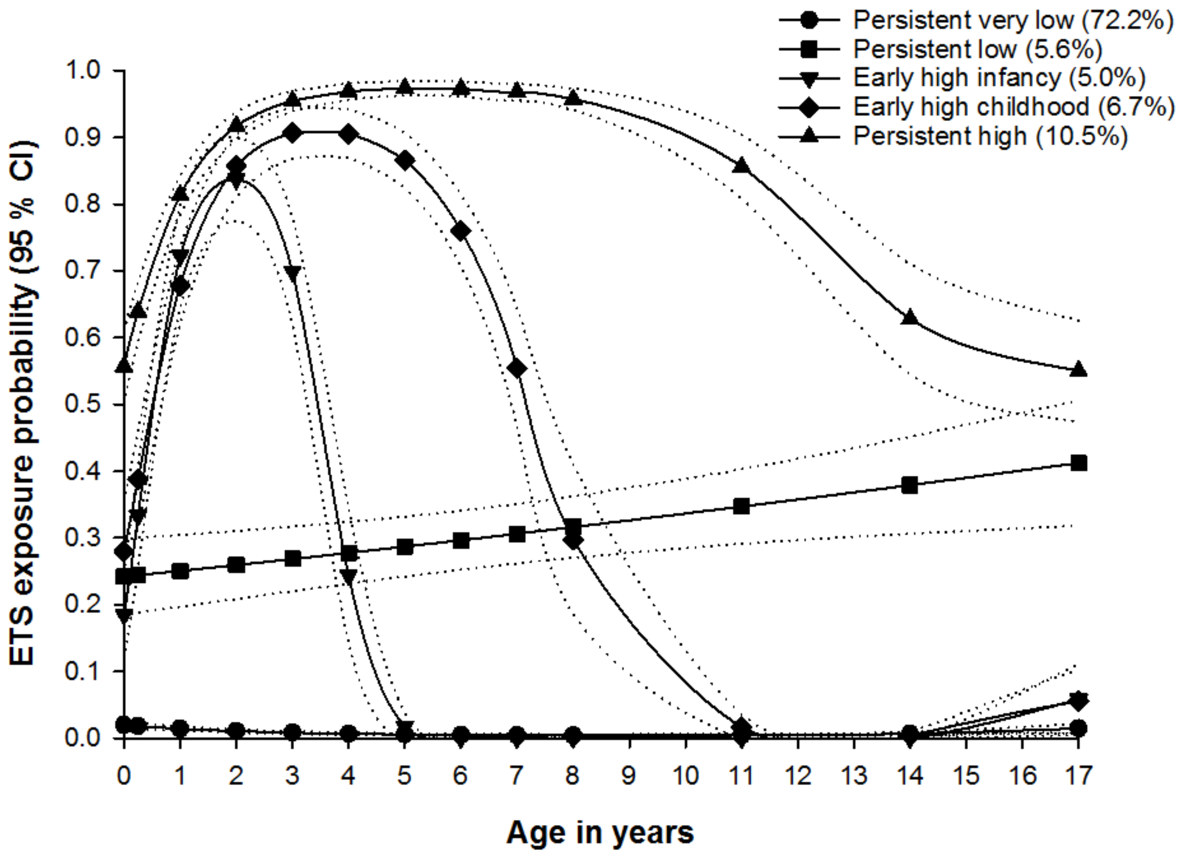

Figure S6. Adjusted odds ratios for association of SHS exposure and asthma at age 17 with longitudinal patterns for extended population of children with asthma data at age 17, but incomplete SHS exposure data. (N=1871) ‡

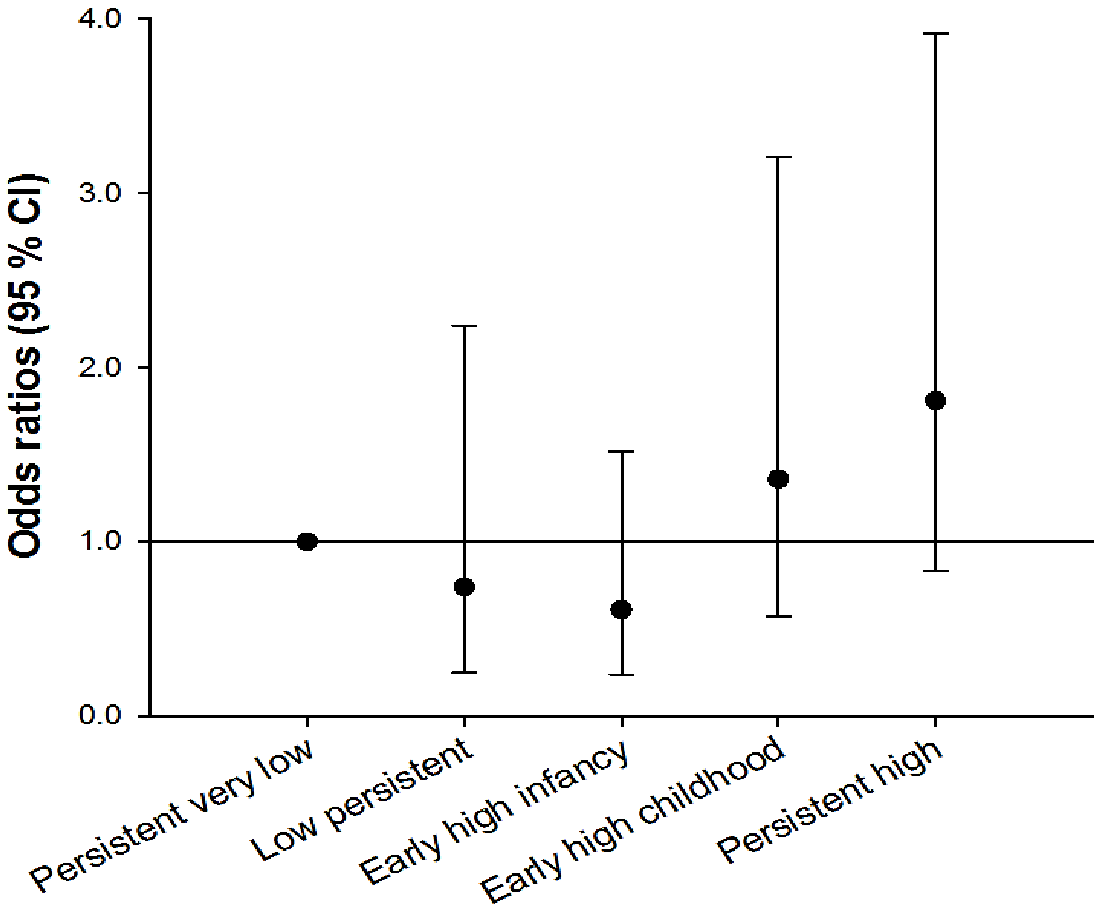

‡ Adjusted for gas cooking at 3 months, overweight at 3 years, presence of pets at 3 months , presence of molds at 1 year , outdoor NO_2 exposure at home address at birth, gender, active smoking, breastfeeding, older siblings at birth, parental atopy, parental education, region and maternal age.

Figure S7. Adjusted odds ratios for association of SHS exposure and asthma until 17, adjusted for time varying confounders defined at age 17 years instead of early life†

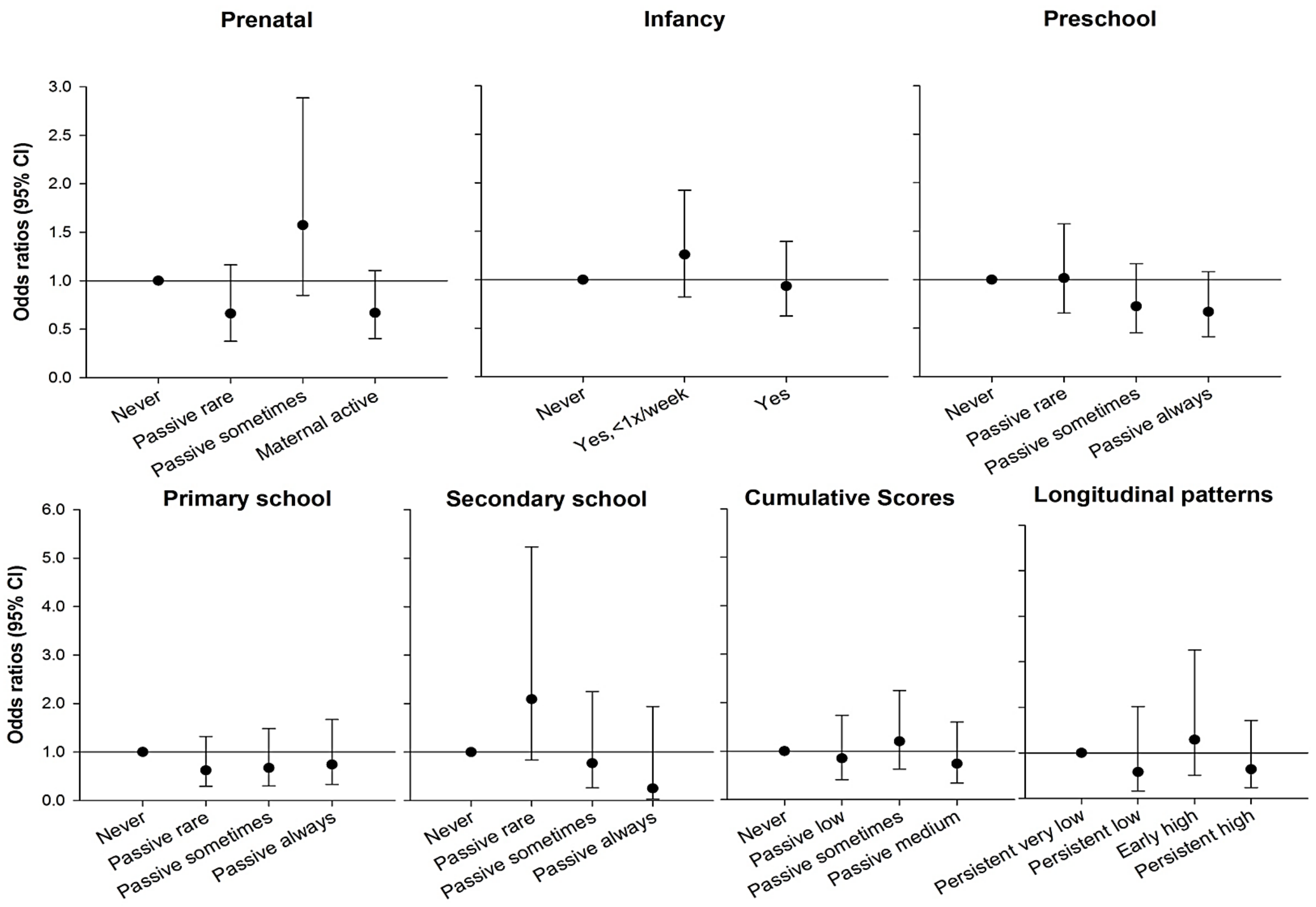

† Associations are overall associations with asthma at ages 4 to 17 years from GEEs for prenatal, infancy and preschool exposure, associations with asthma at age 17 from cross-sectional analyses for primary and secondary school windows. Adjusted for gas cooking, overweight, presence of pets and presence of molds at 17 years , outdoor NO_2 exposure at home address at 14 years, gender, breastfeeding, active smoking, older siblings at birth, parental atopy, parental education, region and maternal age at birth.

Figure S8 Adjusted odds ratios for association of SHS exposure and asthma until 17, for time windows, additionally adjusted for low birth weight*

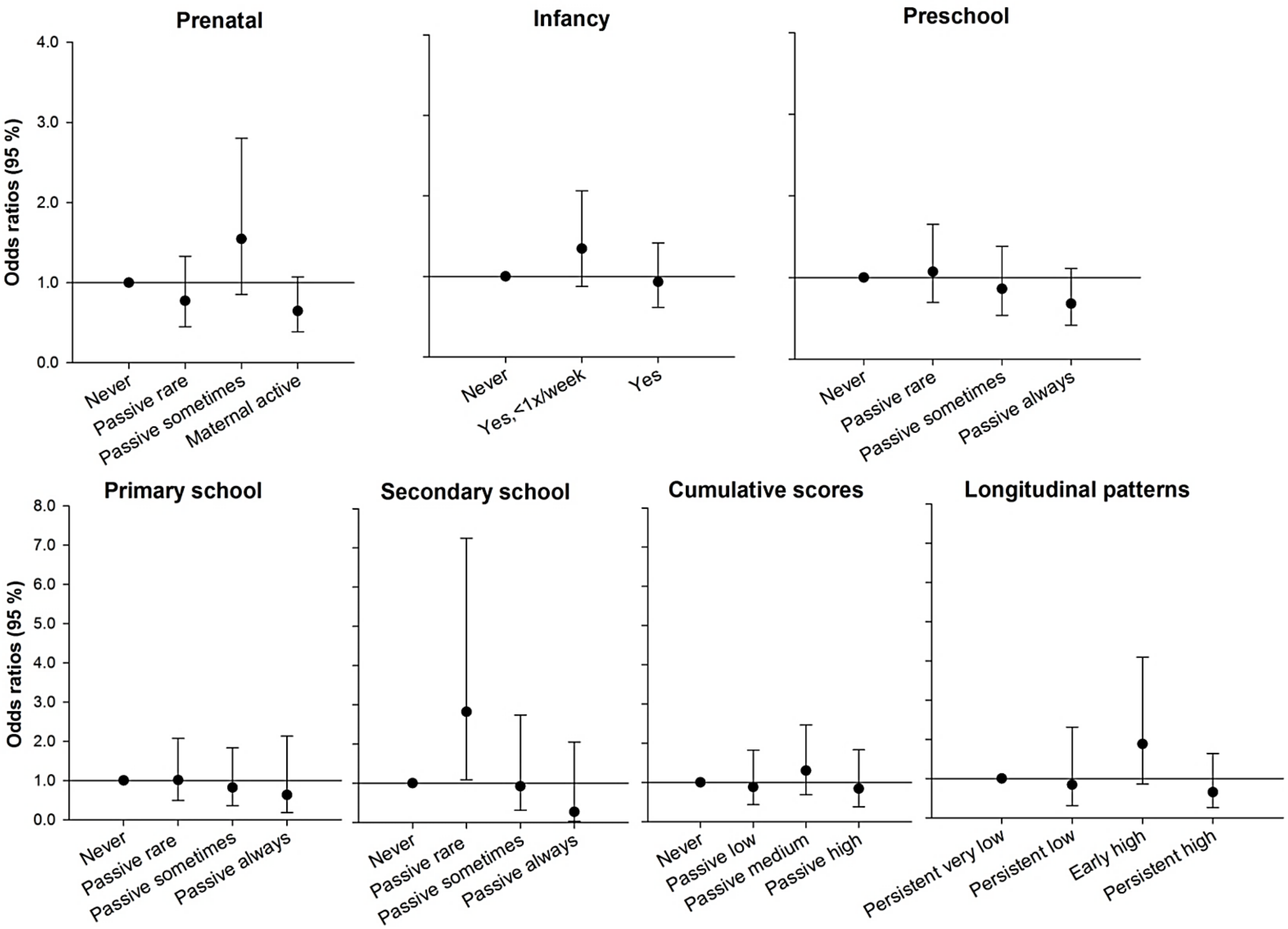

* Associations are overall associations with asthma at ages 4 to 17 years from GEEs for prenatal, infancy and preschool exposure, associations with asthma at age 17 from cross-sectional analyses for primary and secondary school windows. Adjusted for gas cooking, overweight, presence of pets and presence of molds at 17 years , outdoor NO_2 exposure at home address at 14 years, gender, breastfeeding, active smoking, older siblings at birth, low birthweight, parental atopy, parental education, region and maternal age at birth.

Figure S9. Adjusted odds ratios for association of SHS exposure and asthma until age 17 excluding active smokers[+]

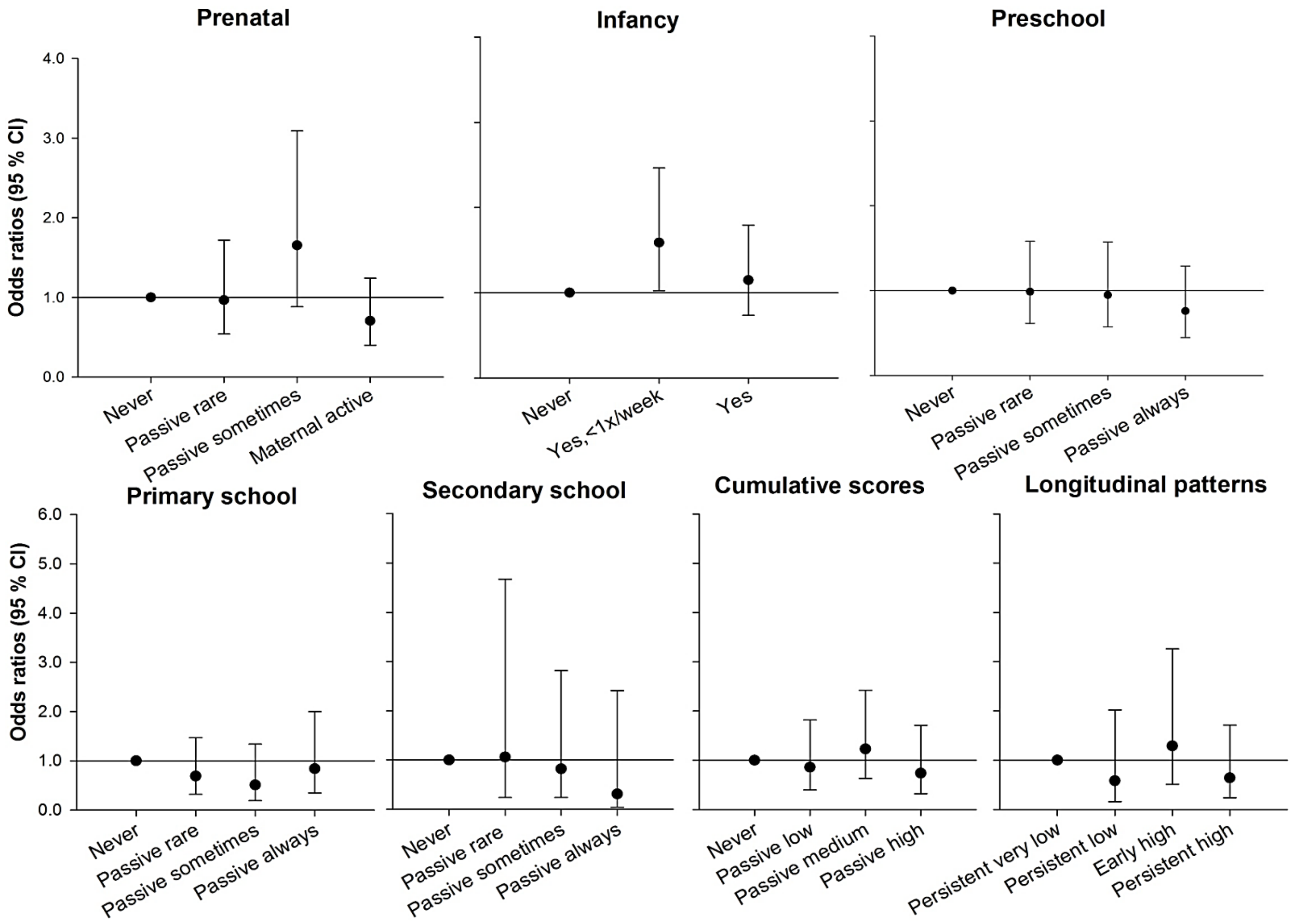

\+ Adjusted for gas cooking at 3 months, overweight at 3 years, presence of pets at 3 months , presence of molds at 1 year , outdoor NO_2 exposure at home address at birth, gender, active smoking, breastfeeding, older siblings at birth, parental atopy, parental education, region and maternal age. Overall longitudinal estimates reported for prenatal, infancy and preschool time windows.

Figure S10. Adjusted odds ratios for association of SHS exposure and asthma until age 17 stratified by atopy preschool time window-specific exposure.

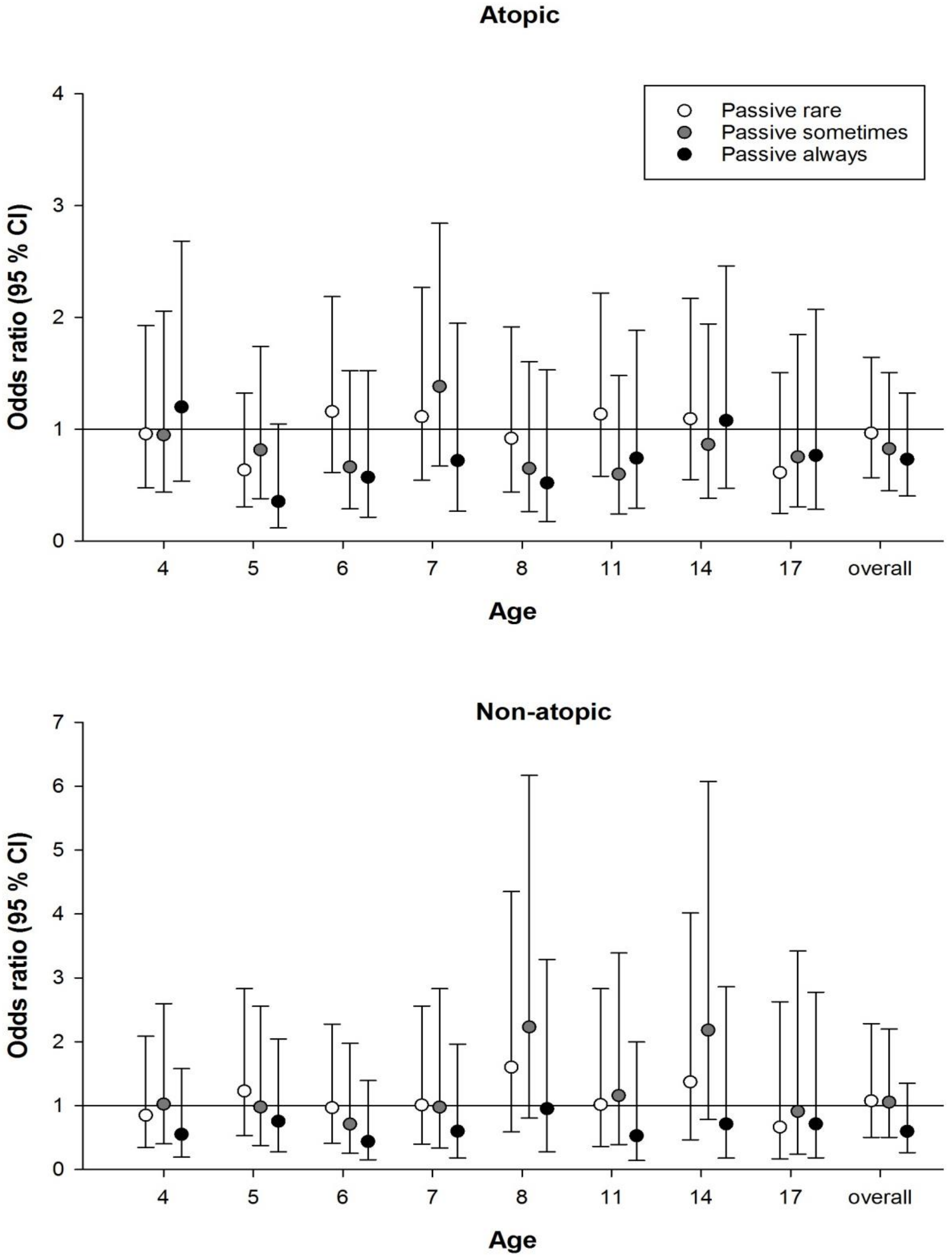

α Adjusted for gas cooking at 3 months, overweight at 3 years, presence of pets at 3 months , presence of molds at 1 year , outdoor NO_2 exposure at home address at birth, gender, breastfeeding, active smoking, older siblings at birth, parental education, region and maternal age.

Reference group= Never exposed

Figure S11. Adjusted odds ratios for association of SHS exposure and asthma until age 17 stratified by presence of pets for preschool time window-specific exposure.

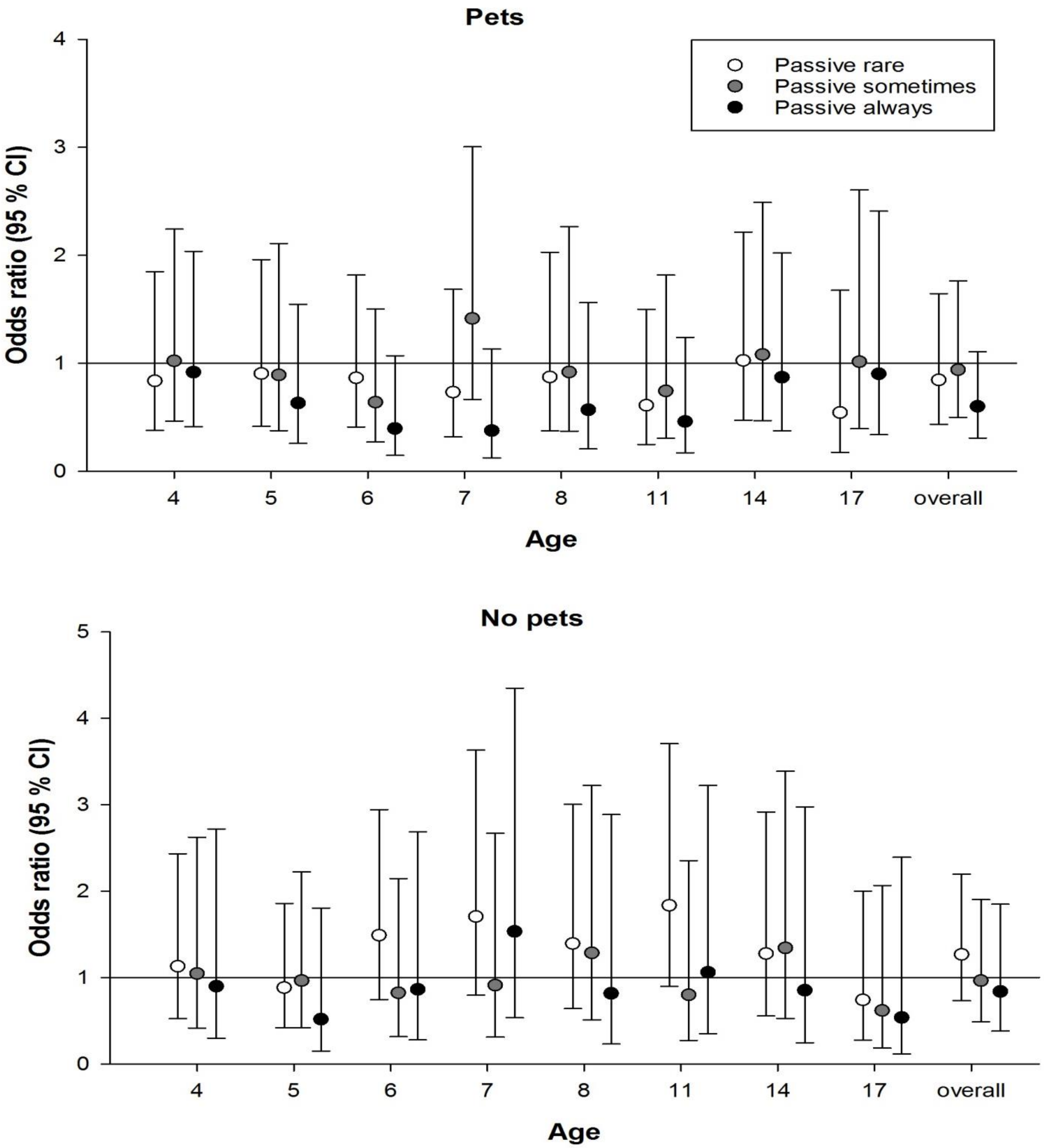

γ Adjusted for gas cooking at 3 months, overweight at 3 years, presence of molds at 1 year , outdoor NO_2 exposure at home address at birth, gender, breastfeeding, active smoking, older siblings at birth, parental education, region and maternal age.

Reference group= Never exposed

Figure S12. Adjusted odds ratios for association of SHS exposure and asthma until age 17 stratified by gender for preschool time window-specific exposure. ϴ

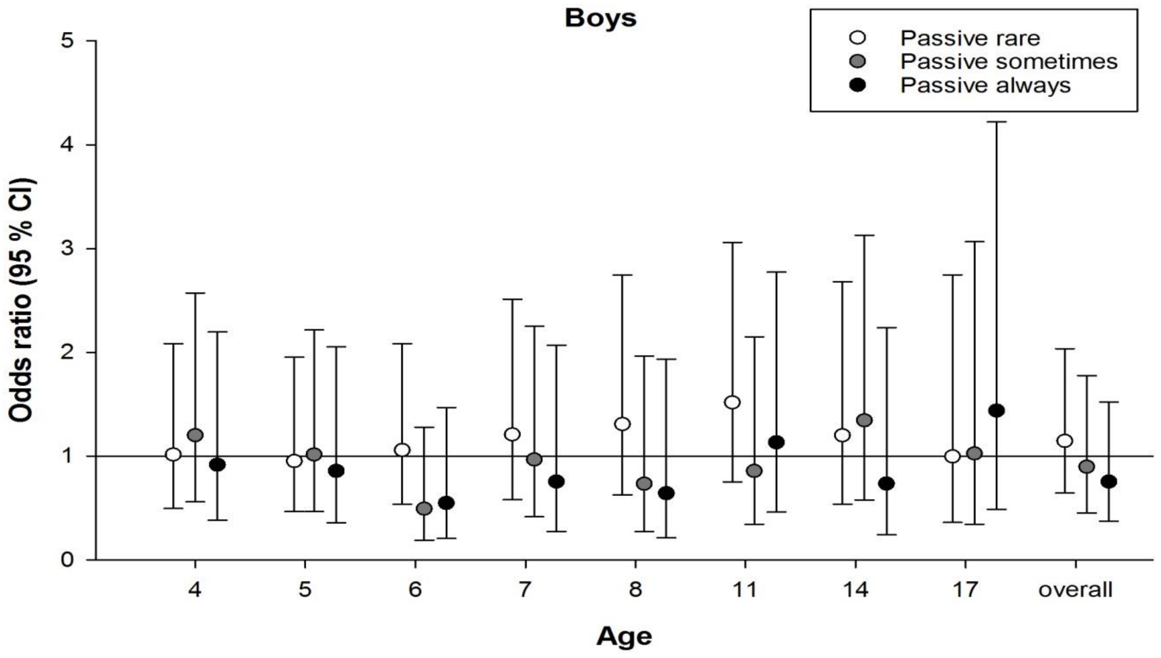

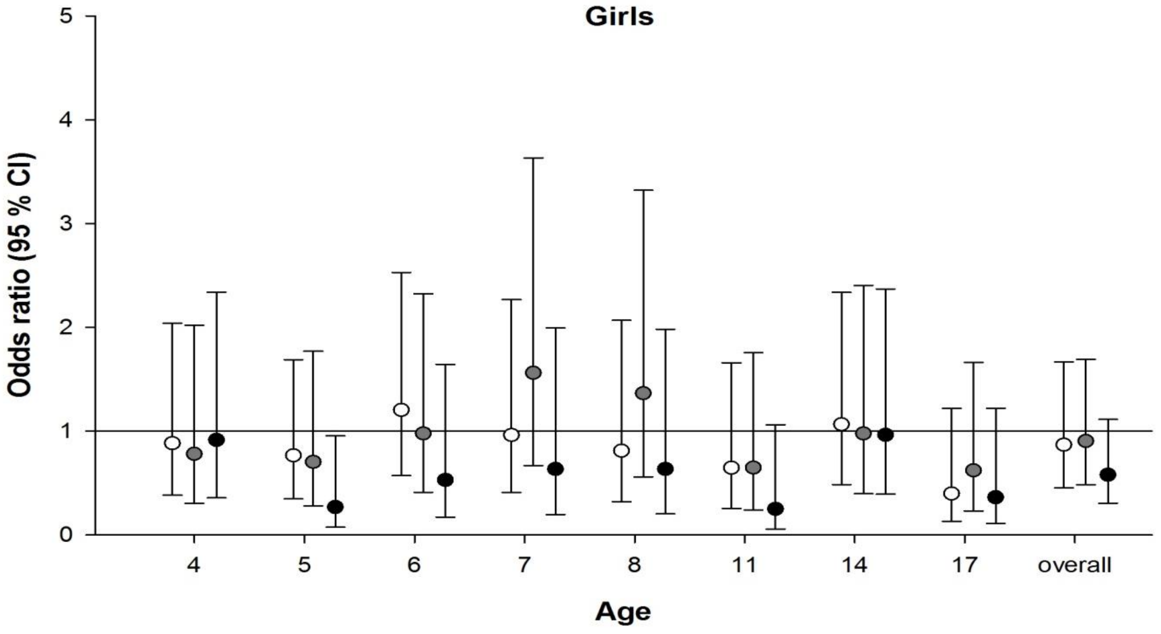

ϴ Adjusted for gas cooking at 3 months, overweight at 3 years, presence of molds at 1 year , outdoor NO_2 exposure at home address at birth, gender breastfeeding, active smoking, older siblings at birth, parental atopy, region and maternal age.

Reference group= Never exposed

Figure S13. Adjusted odds ratios for association of SHS exposure and asthma until age 17 stratified by parental education for preschool time window-specific exposure. β

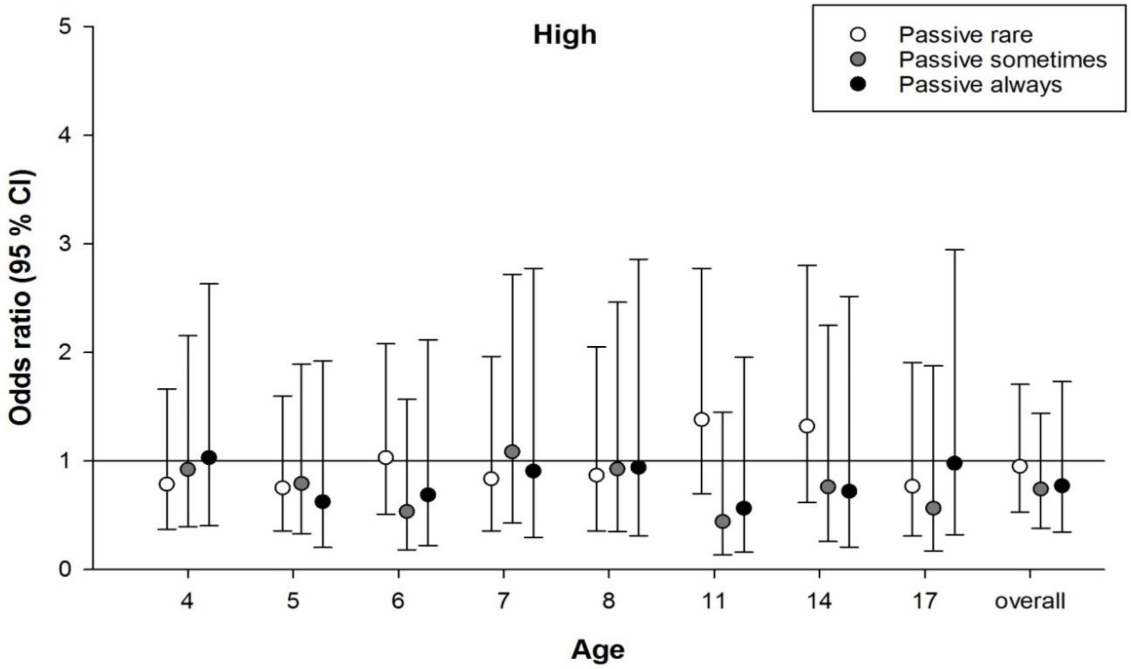

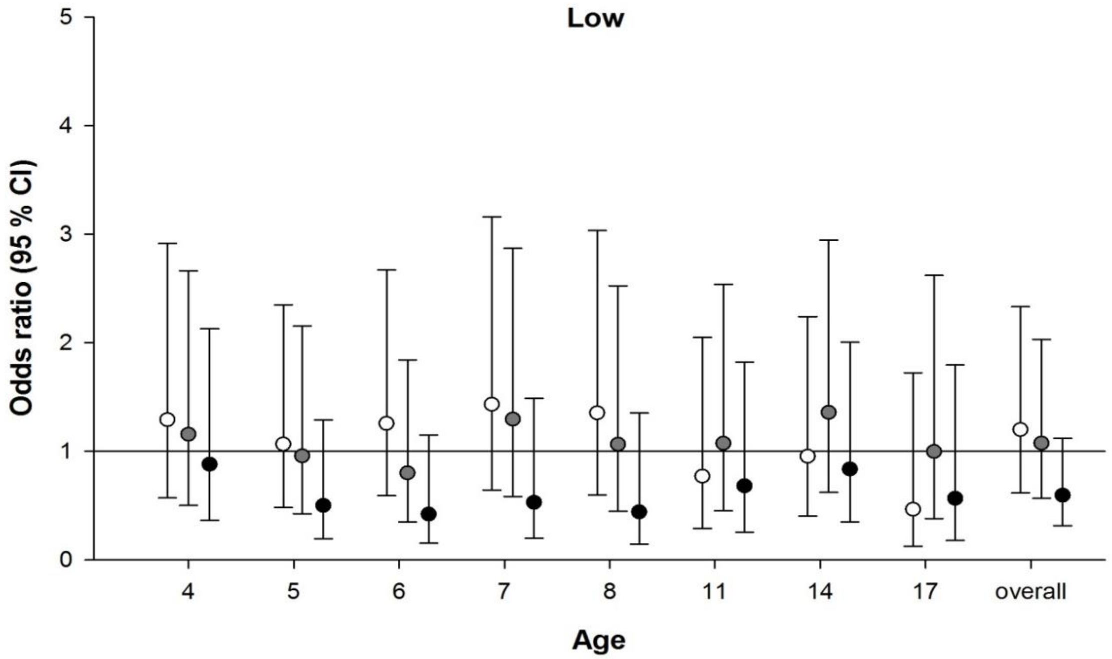

β Adjusted for gas cooking at 3 months, overweight at 3 years, presence of molds at 1 year , outdoor NO_2 exposure at home address at birth, gender breastfeeding, active smoking, older siblings at birth, parental atopy, region and maternal age.

Reference group= Never exposed
